# Emerging Roles of the α-Catenin Family Member α-Catulin in Development, Homeostasis and Cancer Progression

**DOI:** 10.3390/ijms231911962

**Published:** 2022-10-08

**Authors:** Mateusz Gielata, Kamila Karpińska, Tomasz Pieczonka, Agnieszka Kobielak

**Affiliations:** Laboratory of the Molecular Biology of Cancer, Centre of New Technologies, University of Warsaw, 02-097 Warsaw, Poland

**Keywords:** α-catulin, CTNNAL1, catenin, invasion, epithelial-mesenchymal transition, EMT, vascular mimicry

## Abstract

α-catulin, together with vinculin and the α-catenins, belongs to the vinculin family of proteins, best known for their actin-filament binding properties and crucial roles in cell-cell and cell-substrate adhesion. In the past few years, an array of binding partners for α-catulin have surfaced, which has shed new light on the possible functions of this protein. Despite all this information, the molecular basis of how α-catulin acts in cells and controls a wide variety of signals during morphogenesis, tissue homeostasis, and cancer progression remains elusive. This review aims to highlight recent discoveries on how α-catulin is involved in a broad range of diverse biological processes with an emphasis on cancer progression.

## 1. Introduction

Homeostasis in healthy tissues strongly depends on cadherin- and integrin-mediated, cell-to-cell and cell-to-extracellular matrix (ECM) adhesion, respectively [1]. Both types of adhesion are crucial for maintaining tissue architecture and sensing and responding to changes in their environments. Cadherins are transmembrane glycoproteins that mediate calcium-dependent cell-cell adhesion. Through their homophilic binding interactions, cadherins play a role in cell-sorting mechanisms, conferring adhesion specificities on cells. The regulated expression of cadherins also controls cell polarity and tissue morphology. Classical cadherins are located at adherens junctions and are characterized by five homologous repeats at the extracellular domain. In contrast, the intracellular classical cadherin cytoplasmic domain (CCD) binds armadillo family proteins β-catenin (Ctnnb1) and p120ctn (Ctnnd1). The interaction with β-catenin links cadherins to α-catenin and the actin cytoskeleton, whereas p120ctn is involved in cadherin turnover. By regulating contact formation and stability, cadherins play a crucial role in tissue morphogenesis and homeostasis [1].

The adhesion of cells to the extracellular matrix (ECM) is mainly mediated by integrins, which undergo a conformational change upon activation to recruit structural and signaling molecules. Thus, integrins not only mechanically couple the cytoskeleton to the ECM but also transmit molecular signaling cascades to regulate cellular functions in response to extracellular cues [1].

During tissue morphogenesis, wound healing or pathological alterations in diseases like cancer, the ability of cells to rapidly and reversibly change adhesive properties is a key feature. This cell plasticity is driven by the programs of the epithelial–mesenchymal transition (EMT) and mesenchymal–epithelial transition (MET), both of which play essential roles during normal embryogenesis and tissue homeostasis [2]. However, the aberrant activation of these processes can also drive different stages of cancer progression, including invasion, cell dissemination, metastatic colonization, and secondary tumor outgrowth [3]. EMT enables physically connected epithelial cells to disassociate their characteristic classical cadherin/catenin cell-cell contacts, lose their apical-basolateral polarity, and increase expression and activity of integrins displaying leading-edge asymmetry to become motile and mesenchymal-like and capable of degrading the basement membrane. The events occurring during EMT include the downregulation of cytokeratins and E-cadherin, epithelial-specific markers, and an increase in mesenchymal markers, such as fibronectin, N-cadherin, and vimentin. Transcription factors, including Snail1/Snail, Snail2/Slug, Twist, and ZEB1, are well known to be involved in the orchestration of EMT. Cell–cell adhesion and cell–ECM sites contain overlapping functional constituents containing common and distinct proteins. The crosstalk between these adhesion sites is crucial to coordinate cell migration with dynamic interactions between cells. Because both integrins and cadherins associate with the cytoskeleton and many common signaling molecules, it is likely that the cell–ECM and cell–cell adhesion processes mediated by these two types of receptors act in a coordinated manner in regulating cellular functions [4]. Changes in expression or mutations of these proteins, especially cadherins, catenins, and integrins, are frequently associated with diseases ranging from developmental defects to carcinogenesis and metastasis [5,6,7]. It is well established that two significant hallmarks of cancer, namely loss of cell-to-cell adhesion and anchorage-independent growth, are both dependent on cell adhesion molecules. Vinculin and α-catenin are two related proteins that play crucial roles in those processes [4,8]. However, the function of their recently characterized homolog α-catulin is still poorly understood. α-catulin is a protein whose name is composed of “α-cat”, which comes from α-catenin, and “ulin”, which comes from vinculin as it is a homolog of α-catenin protein belonging to the vinculin superfamily. Despite the sequence homology and shared superfamily with α-catenin, α-catulin’s localization and functions appear to differ. Multiple reports describe α-catulin as an important factor contributing to cancer cell migration and invasion; however, the exact molecular mechanism leading to this phenomenon remains unclear. A growing number of reported α-catulin-interacting partners and new connections imply even more complex regulatory functions for this protein. This review aims to highlight recent discoveries emphasizing how α-catulin is involved in the coordination of a network of signals and actin cytoskeleton regulation.

## 2. α-Catulin—A Member of the α-Catenin Family

**Figure 1:**
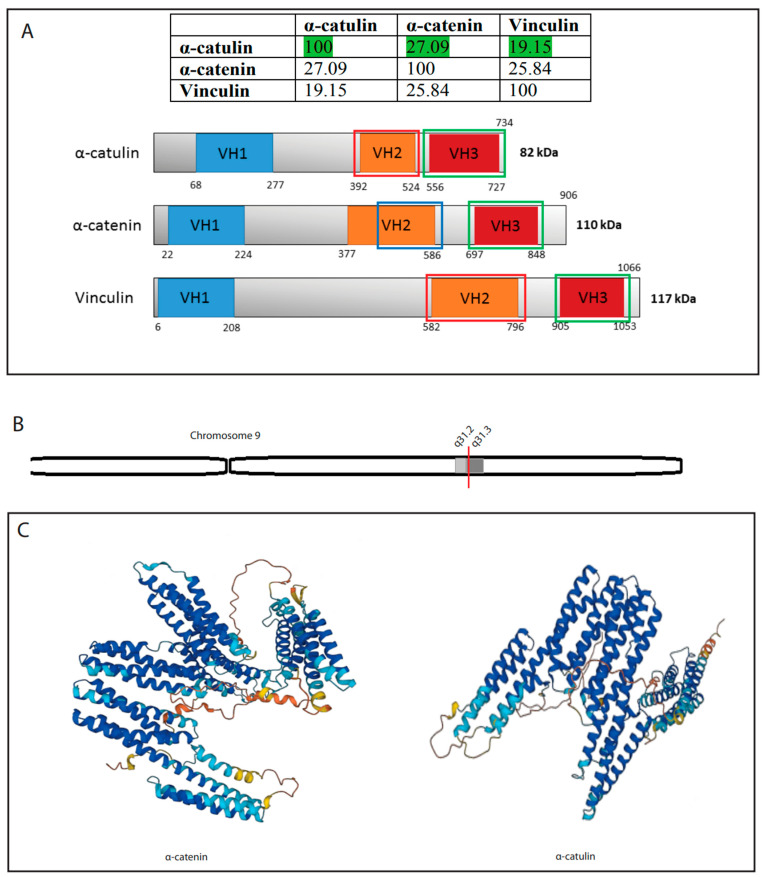
Structural features of α-catulin. (**A**) Table represents amino acid sequence similarities (%) between α-catulin, α-catenin and vinculin. α-catulin shares 27.09% homology with α-catenin and 19.15% with vinculin. (**B**) Schematic representation of α-catulin (CTNNAL1) gene on chromosome 9 locus 31.3. (**C**) Scheme shows the predicted 3D structure of α-catulin and α-catenin protein by AlphaFold.

Whereas all other catenins (β-catenin, plakoglobin and p120 catenin) share relatively high sequence homology and belong to the Armadillo family of proteins, vinculin and α-catenins differ in sequence and structural organization and form the vinculin family [9] together with the recently characterized homolog α-catulin [10]. Although it is well known that α-catenin is necessary for cadherin-catenin-mediated cell-cell adhesion, and vinculin is important for integrin-mediated cell-ECM adhesion and cell-cell adhesion, the function of α-catulin is still not well understood. α-catulin (catenin alpha like 1) protein is encoded by the *CTNNAL1* gene located on chromosome 9 in loci q31–32 (Figure 1B) positions 108,942,569–109,013,522 in a minus-strand orientation, resulting in a base length of 70,954 (https://www.genecards.org/cgi-bin/carddisp.pl?gene=CTNNAL1 (accessed on 5 September 2022)). The protein is 734 amino acids long and weighs 81,896 Da (https://www.uniprot.org/uniprotkb/Q9UBT7/entry (accessed on 5 September 2022)). However, two other alternative splicing isoforms have been described, one with substitution in positions 714–734 [10] and another with missing aa in positions 397–480 [11]. There is no 3D structure for α-catulin that has been deposited in the PDB file. The only available structure is the one predicted by AlphaFold (https://alphafold.ebi.ac.uk/ (accessed on 5 September 2022)) which still has a poor structural prognosis in some locations (Figure 1C). mRNA of α-catulin is widely expressed in the human body. It has been reported to be expressed in the thymus, prostate, testes, ovary, small intestine, colon [12], skeletal muscle, lung, heart, and placenta [10]. The human protein atlas also confirms that α-catulin protein is widely found in the human body, interestingly having the highest score in endocrine tissues, female and muscle tissues (proteinatlas.org). In 2002, Park et al. demonstrated that when using the Blast tool and analyzing the *CTNNAL1* sequence, they reported having high similarity to α-catenin. The BESTFIT similarity alignment of α-catulin with its closest human homolog α-catenin showed 27% identity, and alignment with vinculin showed almost 20% identity (Figure 1A). They also showed that α-catulin is characterized by an extra 16 N-terminal amino acids not present in mammalian α-catenins. α-catulin and α-catenin homology is represented by two blocks; the first homology sequence is between α-catulin residues 18–524 and αN-catenin positions 2–504. The following sequence is a region of 110 amino acids present in α-catenin that is omitted in α-catulin. The second homologous block extends α-catulin residues 525–734 [12]. α-catulin also shows high sequence similarity with vinculin, hence being categorized as a part of the vinculin superfamily of proteins. The homology with vinculin, however, is lower, reaching 21%. The similarity is essentially high in the N-terminal domain of the protein, shown to have putative binding sites for β-catenin, talin, and α-actinin [10]. Amphipathic helices in the C-terminal region corresponding to α-catenin contain potential binding sites for the actin cytoskeleton. This region also contains potential binding sites for ZO-1, the protein important for tight junctions [10,12], another type of intercellular adhesion complex that forms the border between the apical and basolateral cell surface domains in polarized epithelia and controls paracellular permeability. Despite the sequence homology between α-catulin and α-catenin, their subcellular localization pattern is different as shown by Park et al. α-catulin localized to both the membrane-rich (pellet) fraction and the soluble (cytosolic) fraction, whereas α-catenin was found to localize almost exclusively to the membrane-rich fraction. They confirmed those results with two different experiments, one with high-speed fractionation into cytosolic and membrane-rich fractions followed by Western blotting, and the second with Myc-tagged α-catulin (pcDNA Myc:α-catulin) and indirect immunofluorescence. Despite the above-mentioned characteristics of α-catulin, it is still very poorly characterized.

## 3. Binding Partners of α-Catulin

One of the first described interacting partners of α-catulin is Lbc Rho guanine nucleotide exchange factor. Rho guanine nucleotide exchange factor (GEF) functions for the RhoA small GTPase protein [13]. RhoA is inactive when bound to the GDP, but when acted on by the Rho GEFs, GDP can be released, and GTP might be attached, leading to the activation of RhoA. Furthermore, active RhoA can bind to and activate distant effectors or enzymes. Interestingly, in this particular case, RhoA is a major regulator of the cell actin cytoskeleton [14]. One of the GEFs specific for Rho is a DH domain containing Lbc oncogenic product GEF [15,16]. All Lbc Rho GEF forms possess common C-terminal regions following DH domain cassette [17]. Park et al. showed a direct association between Lbc Rho GEF and α-catulin using three independent systems: yeast two-hybrid interaction, direct binding in vitro, and complex formation in mammalian cells. The required site of interaction within the Lbc C-terminal region was mapped to the ∼253-residue IDR (intrinsically disordered region). They also determined that the α-catulin site required for the interaction lies in the N-terminal residues 34–524. Coexpression of α-catulin and wt-Lbc led to increased GTP-Rho formation in cooperative action. This implies that α-catulin is an upstream regulator of Rho. Overall, the authors conclude that α-catulin acts as a scaffold protein for Lbc Rho GEF and facilitates Lbc-induced Rho signals [12,17]. 

**Figure 2:**
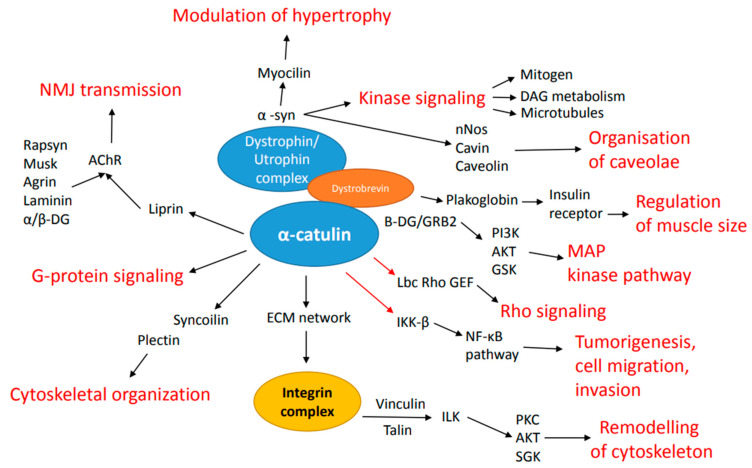
Overview of the function of α-catulin in dystrophin complex. In blue circles, α-catulin and dystrophin complex are shown. The interaction occurs via dystrobrevin, highlighted in orange. Shown are distinct interactors of the complex as well as direct interactors of α-catulin. Enlarged is also integrin complex, having interactions indirectly via ECM and impacting cytoskeleton remodeling. Highlighted in red are key pathways and functions resulting from either interaction of the complex or α-catulin directly.

α-Catulin has also been shown to interact with the dystrophin complex through direct interaction with dystrobrevin in *C. elegans.* This interaction is conserved and also present in mouse skeletal muscles [18]. Dystrophin has been known as a cause of Duchenne muscular dystrophy, yet dystrophin usually functions in protein complexes known as dystrophin-associated protein complex (DAPC) [19]. It had been previously shown that mutations in the CTNNAL1 gene lead to the interruption of DAPC localization near dense bodies [20]. In the above-mentioned publication, the reciprocal action of α-catulin with dystrobrevin was validated by co-immunoprecipitation as well as by mass spectrometry and yeast two-hybrid screen. The authors observed an increase in α-catulin expression levels in the skeletal muscle of dystrophin-deficient mice, where dystrophin-associated protein complex is disassembled, and the link between the costamere and the sarcolemma is absent. To bind α-catulin, dystrobrevin requires a C-terminus as well as an α-helix H2 proximal to the C-terminal region [18]. Similar results have been obtained in other studies. Lyssand et al. showed that the C-terminal domain of dystrobrevin recruits α-catulin to the α_1D_-AR signalosome. Adrenergic receptors (ARs) and G protein-coupled receptors (GPCR) are important regulators of cardiovascular system function. Their function revolves around increasing blood pressure and promoting vascular remodeling. [21]. Sequence analysis revealed that, similar to α-catenin, α-catulin has a putative binding domain for β-catenin; therefore, a group led by Deniz Toksoz took a closer look into this interaction, mapping it to the N-terminal 163 amino acids of the protein [21,22]. When performing co-immunoprecipitation, they noticed that α-catulin indeed co-precipitates with β-catenin, but the amount of α-catulin associated with β-catenin appeared to be smaller than that of α-catenin associated with β-catenin. Given that endogenously in cells, the pool of β-catenin is naturally bound to α-catenin, these results were not surprising. α-catulin might associate with a different fraction of β-catenin than α-catenin does. There might be other pools of β-catenin, such as tyrosine phosphorylated β-catenin, in which protein interactions are altered [21,23,24,25]. Here, the authors additionally proposed the antiproliferative role of α-catulin, as it attenuates cyclin D1 transcription, leading to decreased cyclin D1 protein levels. They also observed that expression of α-catulin had a negative impact on cancer cell colony formation ability, leading to the statement that α-catulin modulates endogenous growth signaling pathways [21,22]. As β-catenin functions at the adherens junctions and also acts in the nucleus after stabilization of a pool of β-catenin in response to the upstream Wnt signals, it is crucial to further investigate the catulin-β-catenin interaction. Another α-catulin interacting protein was reported in the publication by Wiesner et al. in 2008. It was shown that α-catulin can modulate the NF-κB pathway by binding to IKK-β [21,26]. The NF-κB pathway plays a pivotal role in a variety of biological processes like innate and adaptive immune responses, tissue differentiation and apoptosis [21,27,28]. The targets of NF-κB include its own inhibitors IκBα and IκBβ [21,29]. Different extracellular stimuli activate the IκB kinases IKK-α and -β, which phosphorylate IκBα, which results in the degradation of IκBα and translocation of NF-κB to the nucleus [21,30]. Wiesner et al. provided evidence that α-catulin binds to IKK-β by immunoprecipitation. Moreover, they limited the interaction site in α-catulin to its C-terminal 87 amino acids. As α-catulin binds to IKK-β in the C terminus and to Lbc Rho GEF in the N-terminus, the authors claim that it may allow simultaneous stimulation of both pathways, being a bridge between those two. As there is evidence that both the NF-κB and RhoA signaling pathways play multiple roles in tumorigenesis, cell migration, invasion, and escape from apoptosis, α-catulin, as a linker of those two pathways, might serve as a crucial clinical target [21,26,31]. The interaction of α-catulin with Lbc, dystrophin complex and other proteins and resulting pathways activation have been represented in Figure 2.

Finally, α-catulin has been described as an interactor protein of human NEK1 protein kinase. NEK kinases are involved in regulating diverse cellular processes like the cell cycle, mitosis, cilia formation, and the DNA damage response and the etiology of polycystic kidney disease (PKD). α-catulin has been described as one of the 11 new binding proteins of NEK1. Moreover, it has been proven to interact with both regulatory and kinase domains (NRD and NKD) [32]. Interestingly, aberrant expression of NEKs appears to be involved in the initiation, maintenance, progression and metastasis of cancer and is associated with a poor prognosis [33]. A better understanding of NEK1 kinase interaction with α-catulin may lead to more successful clinical trials of NEK inhibitors. 

## 4. α-Catulin and Its Function during Development

The plethora of interacting proteins indicates that α-catulin may play essential roles in various vital regulatory processes. Thus far, the important role of α-catulin has been shown in the process of neurulation during mouse development [34], where cell-cell and cell-ECM interactions are constantly under remodeling to enable proper architecture and function of forming tissues. The actin-cytoskeleton and actomyosin contractility integrated at the cell-cell and cell-ECM adhesions cooperatively are crucial to shape the cells and tissues [35,36,37]. The adherens junctions are required for the transmission of force across an epithelium, and the actomyosin cortex, which spans the apical surface of an epithelium, transitions between elongation and active state of actin nucleation while still attached to the adherens junction, allowing for apical constriction, which is crucial, for example, during neurulation [37,38,39]. It is important that actomyosin machinery is located at the right place and time to generate the required force to pull the neural folds together [40]. Interestingly, α-catulin was shown to participate in the apical actomyosin network regulation by serving as a scaffold protein that may be important for properly directing Rho family GTPase signaling during neurulation. α-catulin-deficient mice show neural tube (NT) closure defects. They are embryonically lethal with massive disorganization of their neuroepithelium, extra bending, absence of apically localized actin filaments, nestin and phosphorylated myosin, and inappropriate basement membrane assembly due to very low expression of its components: laminin and fibronectin. The neuroepithelium of α-catulin deficient mice lack apically localized actin filaments and P-Mlc, which typically correlate with proper Rho-dependent cell constriction. In vitro studies performed in a three-dimensional model of MDCK cells showed that α-catulin is localized specifically at the apical parts of cells membranes and is important for proper cell polarization, organization of actomyosin cytoskeleton, stabilization of intercellular junction as well as distribution of active Rho A. Taken together, data collected both from in vivo mouse model and in vitro 3D studies indicated a pivotal role of alpha-catulin protein in neurulation during embryonic development, as it can act as a scaffold for RhoA in apical parts of cells, which results in correct spatial activation of downstream myosin to influence actin-myosin dynamics and the stability of cell-cell junctions, which allows generating the appropriate tension needed for the apical constriction of cells and proper bending of the neural plate [34]. 

## 5. Role of α-Catulin in Homeostasis

In the last decade, numerous studies have also demonstrated the importance of α-catulin in the maintenance of tissue homeostasis. A-catulin was reported to play potential functions in hematopoietic stem cells (HSCs), bronchial epithelium, muscles and intestine [18,20,41,42,43,44,45]. In hematopoietic stem cells, α-catulin is expressed only in a specific population of 0.02% of bone marrow hematopoietic cells. Generation of a mouse model with green fluorescent protein (GFP) knocked-in into the α-catulin locus allowed to show that α-catulin together with c-kit marks the population of cells that possess the long-term multilineage reconstitution ability of bone marrow after irradiation [41]. In addition, the distribution of α-catulin^+^ c-kit1^+^ cells indicates that HSCs are more common in the central marrow than near the bone surface [41,42]. Even though α-catulin proved to be a great marker for HSC visualization in the bone, the exact function of this protein in those cells was not established.

Furthermore, high expression of α-catulin was also detected in bronchial epithelium under ozone-stressed conditions. Results from this study suggest that elevated α-catulin expression may be a protective response aimed at maintaining airway epithelial integrity [43].

Moreover, in neuromuscular junctions, dystrobrevin utilizes α-catulin for proper neurotransmitter receptor (AChR) clustering on myotubes, indicating its important role in a synaptic machinery organization [44]. As an anchor protein that locates potassium channels and neurotransmitter receptors in specific nanodomains, α-catulin plays a key role in the physiological processes related to the neurosecretion as well as excitation of neurons and muscles. Dysfunction of this important protein may be linked to muscular and neurological disorders [20,44]. It has also been reported that α-catulin ortholog is a critical cytoskeletal regulator in C.elegans, crucial for the proper localization of calcium-dependent potassium channels in both neurons and muscles. In muscles, α-catulin, via the dystrophin complex, binds the calcium-dependent potassium channels near L-type calcium channels. In turn, in neurons, α-catulin controls the localization of the potassium channels independently of the dystrophin complex [20,21,46]. The interaction with dystrophin complex seems to be the best characterized so far for α-catulin. 

Recent studies performed on Chinese patients with Hirschsprung disease revealed that α-catulin can be attributed to genetic factors or gene-gene interaction networks responsible for enteric neuronal dysfunction [45]. Interestingly, catulin expression was observed in the enteric innervation of newborn mice [34]. 

## 6. α-Catulin in Cancer Invasion and Metastasis

Even though α-catulin is overall a very poorly described protein, its participation in cancerogenesis and influence on cancer cell invasion and metastasis has been reported and researched in many papers. Both structural and signaling functions of α-catulin may play a role in cancer progression. As mentioned above, α-catulin in the N-terminal region contains binding sites for β-catenin, talin, α-actinin, and the actin cytoskeleton. This suggests that it may function as a cytoskeletal linker protein that is able to modulate cell migration [8]. Cell migration is a process that plays a pivotal role in carcinogenesis and participates in the metastasizing of cancer cells. Metastasis is a complex phenomenon that occurs in all types of cancers and is responsible for death [47]. It is based on the fact that cancer cells escape from the primary tumor, migrate, enter the lumen of blood and lymphatic vessels and reach distant organs, where they can repopulate the tumor mass [48,49]. Cancer cells need to acquire a mesenchymal phenotype in the process called epithelial-to-mesenchymal transition (EMT) [50]. EMT is a phenomenon where cells downregulate proteins involved in apical cell-cell contact and adherence junction formation, such as E-cadherin and α-catenin, and start upregulating proteins specific for mesenchymal features of the cell, such as N-cadherin and vimentin, which results in the enhanced motility of the cells. This switch between relatively stable cell-cell contacts and an increase in motility is crucial for cancer invasion [51]. It has been observed that when α-catenin, a cell-cell junction protein, is conditionally lost in the epithelium, cells begin to demonstrate increased proliferation rates, migrative properties, and the squamous cell carcinoma (SCC) phenotype [52]. Using microarray analysis to compare mouse α-catenin cKO keratinocytes, which failed to form cell–cell junctions, and WT epithelial cells, it was observed that α-catulin is highly upregulated in the cells with increased motility and mesenchymal phenotypes [52]. This data suggested the participation of α-catulin in cancer progression and was further investigated by our group in a model of human head and neck squamous cell carcinoma (hHNSCC), which is a very aggressive tumor type and accounts for more than 450,000 malignancies diagnosed each year. Despite new treatment options, patients are still faced with a very high rate of recurrence and metastatic disease, with a 5-year survival rate of only 50% [53,54,55]. It was shown that α-catulin is upregulated in the metastatic cells in the xenotransplant in vivo model and also in vitro in the hSCC (human squamous cell carcinoma) cell line after EMT induction. Moreover, α-catulin is highly expressed at the invasion front and in migrating, metastatic streams of cells in human samples of HNSCC and in higher grades of tumor samples when compared with normal mucosa epithelium [56]. Most importantly, ablation of α-catulin in hSCC cells decreased the ability of these cells to migrate and invade in vitro and decreased their metastatic potential in vivo [56]. Given that the expression of α-catulin not only correlates with tumor grade, but also appears to be involved in the regulation of the invasive character of the HNSCC cells, it suggests that α-catulin may represent a novel yet critical mediator of oral cancer progression. As this type of cancer usually spreads locally, utilizing collective migration, α-catulin could be important for spatiotemporal fine-tuning of Rho GTPases within a group of cancer cells to control divergent cell-cell and cell-ECM adhesion as well as cytoskeletal functions to achieve cellular coordination and mechanocoupling. This is one of the options that will require further testing to better understand the role of catulin in the process of HNSCC invasion. As α-catulin expression and function correlated with the early onset of hSCC cell invasion, our group used the human α-catulin promoter fragment driving GFP expression to develop a reporter system. This unique system, for the first time, allowed us to isolate in vivo a small population of invasive cells at the human tumor invasion front [57]. After verifying the reporter system, we showed that cells highly expressing GFP driven from α-catulin expression localize at the invasion front in a spheroid model of hSCC cells. Additionally, this system marked the cells with higher migratory, invasive, and tumorigenic potential in vitro in the 3D model. Cells highly expressing α-catulin were also observed in a small population of invasive cells at the tumor front in the in vivo model of head and neck squamous cell carcinoma. Expression of GFP under α-catulin promoter correlated with the loss of an epithelial marker, E-cadherin expression, indicative of ongoing EMT. The reporter system allowed for isolation and transcriptional characterization of those highly invasive cells, providing a list of deregulated genes that are involved in cellular movement, ILK and integrin signalling, as well as axonal guidance signalling [57]. This functional genomic study of the purified population of invasive cells revealed enrichment in genes involved in cellular movement and invasion, providing a molecular profile of HNSCC invasive cells. Interestingly, this profile overlapped partially with the expression of signature genes related to partial EMT available from single-cell analysis of human HNSCC specimens [58]. This comparison strengthens the idea that α-catulin in this type of cancer might be important for spatiotemporal regulation of Rho GTPases within a group of cancer cells to control dynamic plasticity and crosstalk between cadherin-mediated cell-cell contact and integrin-dependent cell-ECM adhesion, which is crucial during collective invasion and migration. Further research on catulin revealed that its role in cancer progression is not only limited to HNSCC specifically, which utilizes collective invasion for local spread. It was recently published that α-catulin is also expressed in human breast cancer samples and triple-negative breast cancer cell lines, and its expression correlates with tumor progression [59]. Breast cancer is now the most common cancer worldwide [60], and the worst outcome is presented by triple-negative breast cancer [61]. Knockdown of α-catulin in triple-negative human breast cancer cell lines MDA-MB-231 and HCC1806 revealed a decrease in the invasion capability of those cells in 3D spheroid model assays [59]. The use of a catulin-GFP-promoter-based reporter system in a 3D spheroid model of triple-negative breast cancer cell lines showed that the most invading cells co-express α-catulin and known EMT marker vimentin. Transcriptional profiling of GFP-positive cells isolated from tumors that formed after injection of a catulin-GFP triple-negative breast cancer cell line disclosed the list of deregulated genes involved in cellular movement and invasion and, interestingly, migration of endothelial cells [59]. Top pathways deregulated in the α-catulin GFP + cells involved epithelial adherens junction signaling and remodeling of epithelial adherens junctions. Special attention was paid to genes involved in the vasculature, as it was observed that tumor areas enriched in GFP+ cells presented visible dense vasculature. Surprisingly, some cells highly expressing GFP co-expressed MCAM (CD146), an endothelial marker but also a cellular surface receptor of different ligands, are actively involved in signaling in the numerous physiological and pathological processes involving metastases of different cancer types. Cells highly expressing GFP and co-expressing MCAM formed vasculogenic structures resembling vessels. This suggests that α-catulin marks highly invasive breast cancer cells that are characterized by increased plasticity and might participate in the process of vascular mimicry, allowing cancer cells to metastasize [59]. In addition, ablation of α-catulin in the in vivo model resulted in decreased tumor size and decreased stemness potential of cancer cells with lowered expression of CD44, which is known to be enriched in breast cancer (BC) stem cells [59]. These data implicate that α-catulin might play an important role in cancer type-specific tumor-microenvironment interplay. Moreover, it may be involved in the inflection of adhesive properties of tumor cells. The possible mechanism of increased α-catulin expression in invasive cancer cells might be explained by the research performed by Cassandri et al. [62]. They showed that zinc-finger protein 750 (ZNF750) is a negative regulator of the migration and invasion of breast cancer cells. It functions as a repressor of a prometastatic transcriptional program. This transcriptional program was shown to express genes that are involved in focal adhesion and extracellular matrix interactions with an emphasis on CTNNAL1. They showed that the expression of CTNNAL1 and LAMB3 contradictorily correlated with ZNF750 expression in a breast cancer model. ZNF750 recruits epigenetic modifiers KDM1A and HDAC1 to the promoter region of the α-catulin gene, which affects histone marks and trans activates these genomic sites. Additionally, they also showed gene expression analysis in cancer patient datasets that indicated ZNF750 and its targets to be negative prognostic factors in breast cancer [62]. In 2011, Fan et al. published a paper confirming the previously described participation of α-catulin in tumorigenesis. They showed that α-catulin expression is elevated in oral cancer cells versus normal cells. They also found that the knockdown of α-catulin resulted in the accumulation of cell populations in S and G2/M cell-cycle phases with decreased cyclin A and cyclin B1 expression. α-catulin knockdown induced cellular senescence as the major phenotype of cell death in two oral cancer cell lines, OC2 and A549. In patients, α-catulin expression correlated with tumor size, whereas α-catulin knockdown supressed tumorigenicity in xenograft models. Knockdown of α-catulin in cancer cells bearing either wild-type or mutant p53 was sufficient to trigger DNA damage response and eventually induce cellular senescence in vitro [63]. In addition to structural functions and regulation of actin cytoskeleton during cancer invasion and migration, α-catulin enhances cancer metastasis by influencing signaling pathways. Liang et al. showed that α-catulin expression correlates with the cell invasiveness potential in vitro and metastatic potential in vivo. It occurs via an ILK/NF-κB/integrin network where α-catulin directly interacts with ILK, which in turn activates the ILK/Akt/NF-κB signaling pathway and upregulates fibronectin and integrin α_v_β_3_. α-catulin as an integrin signaling adaptor might play a pivotal role in regulating integrin-mediated cellular functions via binding to ILK [64]. Later, the same scientific group focused on the participation of α-catulin in cancer stemness and EMT. They found that cells overexpressing α-catulin have genes such as *FGF2*, *BMI1*, *ALDH1A3*, *POU5F1* and *NANOG* upregulated. Additionally, high expression of α-catulin was required to maintain stemness in a lung cancer model, and klf5 was indicated as a new interacting protein that plays an important role in stemness maintenance by cooperating with α-catulin to enhance the transcription of *POU5F1* and *NANOG*. Knockdown of klf5 in cells overexpressing α-catulin abolished the sphere formation capacity. α-catulin not only interacts with klf5 but also protects this protein by blocking the WWP1-mediated proteasomal degradation of KLF5 [65]. The participation of α-catulin in cancer cell migration and invasion has also been proven in a melanoma cancer model. Kreiseder et al. showed that α-catulin is highly expressed in melanoma cells, resulting in reduced E-cadherin and increased N-cadherin expression. Upregulation of α-catulin promotes expression of EMT markers Snail/Slug and Zeb1/2; in addition, α-catulin regulated PTEN and RKIP, inhibitors of the NF-κB pathway. They also found MCAM, plakoglobin, and occludin to be altered in α-catulin-deficient cells. Their results further confirmed that α-catulin is not only responsible for the downregulation of E-cadherin but is also required for melanoma invasion by the upregulation of MMP 2 and 9 and the activation of ROCK/Rho [66]. They further studied the role of α-catulin and, in 2015, published a paper showing that α-catulin is responsible for the chemoresistance of melanoma cells to cisplatin. This reduction in cisplatin-mediated apoptosis of melanoma cancer cells is due to the fact that α-catulin is responsible for NF-κB, AP-1 activation and ERK phosphorylation, and, in the case of knockdown of α-catulin, the cisplatin-mediated apoptosis was shown to be enhanced [67]. 

## 7. Conclusions

α-catulin, together with vinculin and α-catenins, belongs to the vinculin family of proteins, best known for their actin-filament binding properties and crucial roles in cell-cell and cell-substrate adhesion; however, despite sequence homology, α-catulin seems to have independent roles. α-catulin has been shown to be important in inflammation, apoptotic resistance, cytoskeletal reorganization, senescence resistance, cancer progression, and EMT. Multiple binding proteins of α-catulin revealed in recent years suggest a molecular hub function, integrating a cytoskeleton with a number of signaling pathways. Unfortunately, the molecular mechanisms of α-catulin action are still poorly characterized and need further investigation, especially in the field of cancer progression.

Increased α-catulin expression was observed in the invading front of squamous cell carcinoma, and its depletion led to decreased invasion and metastasis in a xenograft transplant mouse model. α-catulin was also reported to be upregulated in a highly invasive non-small cell lung cancer cell line, as well as in breast and prostate cancer. Despite multiple reports describing α-catulin as an important factor contributing to cancer cell migration and invasion, the exact molecular mechanism leading to this phenotype remains unclear.

As α-catulin depletion was shown to have a strong effect on RhoA signaling and the actomyosin cytoskeleton arrangement, it will be crucial to further investigate the role of α-catulin in spatial RhoA distribution during cell migration. Further experiments encompassing mass spectrometry are currently underway in our laboratory in order to identify potential α-catulin interaction partners contributing to the front-rear stabilization of migrating cancer cells. 

A question also remains about potential α-catulin and cadherin interactions. The role of α-catulin in the process of EMT and the switch between relatively stable cell-cell contacts and an increase in motility, which is crucial for cancer invasion, is of great interest. Catulin expression was reported to be upregulated when α-catenin, a cell-cell junction protein, was conditionally lost in the epithelium, which was accompanied by an increased proliferation rate and migrative properties. Therefore, further investigations at the structural, cellular, and functional levels are also needed to understand the exact sequence of molecular interactions and conformational changes operating between the cadherin/β-catenin/α-catenin complex and α-catulin and F-actin and tension-dependent remodeling of cell-cell adhesion. 

An analysis of α-catulin dynamics using high-resolution live imaging should help us to map α-catulin’s localisation and interactions in time and space. These could bring us closer to solving how α-catulin orchestrates adhesion and the actin cytoskeleton.

As α-catulin is broadly expressed and plays multiple physiological functions both during development and adult life, direct therapeutic strategies towards silencing its gene may not be applicable. On the other hand, targeted disruption of signaling pathways originating or ending at α-catulin may be a more promising therapeutic target.
